# Enhancement of InSe Field-Effect-Transistor Performance against Degradation of InSe Film in Air Environment

**DOI:** 10.3390/nano11123311

**Published:** 2021-12-06

**Authors:** Yadong Zhang, Xiaoting Sun, Kunpeng Jia, Huaxiang Yin, Kun Luo, Jiahan Yu, Zhenhua Wu

**Affiliations:** 1 Key Laboratory of Microelectronics Device & Integrated Technology, Institute of Microelectronics Chinese Academy of Sciences, Beijing 100029, China; zhangyadong@ime.ac.cn (Y.Z.); jiakunpeng@ime.ac.cn (K.J.); luokun@ime.ac.cn (K.L.); yujiahan@ime.ac.cn (J.Y.); 2 School of Information Engineering, Hebei University of Technology, Tianjin 300401, China; sunxiaoting@ime.ac.cn; 3 University of Chinese Academy of Sciences, Beijing 100049, China

**Keywords:** InSe, field effect transistors, degradation, hysteresis

## Abstract

The degradation of InSe film and its impact on field effect transistors are investigated. After the exposure to atmospheric environment, 2D InSe flakes produce irreversible degradation that cannot be stopped by the passivation layer of h-BN, causing a rapid decrease for InSe FETs performance, which is attributed to the large number of traps formed by the oxidation of 2D InSe and adsorption to impurities. The residual photoresist in lithography can cause unwanted doping to the material and reduce the performance of the device. To avoid contamination, a high-performance InSe FET is achieved by a using hard shadow mask instead of the lithography process. The high-quality channel surface is manifested by the hysteresis of the transfer characteristic curve. The hysteresis of InSe FET is less than 0.1 V at V_d_ of 0.2, 0.5, and 1 V. And a high on/off ratio of 1.25 × 10^8^ is achieved, as well relative high I_on_ of 1.98 × 10^−4^ A and low SS of 70.4 mV/dec at V_d_ = 1 V are obtained, demonstrating the potential for InSe high-performance logic device.

## 1. Introduction

Recently, increasing attention has been attracted by two-dimensional (2D) materials, including transition metal dichalcogenides (TMDs), Ⅲ-Ⅵ layered semiconductors (InSe, In_2_Se_3_), black phosphorus and graphene, for their potential applications in photo electronic devices, field effect transistors (FETs), integrated circuits and three-dimensional integrated circuit (3D-IC) [1,2,3,4,5]. With the rapid development of the semiconductor process, silicon-based semiconductor materials will not be able to meet the requirements of transistor size reduction in the foreseeable future, especially when the performance of FETs determines the performance of integrated circuits [6]. Conventional high mobility channel materials like InGaAs, Ge also encounter great challenges of strong quantum effect in nanometer scale [7,8], which makes them gradually lose the opportunity in advanced technology node even regardless of the process difficulties. As promising alternative materials for current silicon-based semiconductor devices, the unique layered structure of two-dimensional materials has excellent electrical and optical properties, which makes 2D materials suitable candidates for compact, ultrathin, and high-performance next-generation logic nanodevices [9,10,11]. InSe, which belongs to the family of metal chalcogenide layer semiconductors, is an attractive material in the field of electronics because of its direct and moderate band gap of 1.26 eV of monolayer, ultra-high mobility of 1000 cm^2^·V^−1^·s^−1^ at room temperature, which can be further optimized by strain engineering [12]. Compared with graphene, which is of high mobility but vanished band gap, InSe is more suitable as the channel material for FETs [13]. However, the excellent performance of InSe can be easily hidden by oxidation and impurities absorption when it is exposed to ambient environment and even when the InSe FET is fabricated. In practical applications, this can seriously affect the performance and stability of InSe FETs. To obtain high-performance InSe FET, many passivation layers are chosen to cap on the channel surface, such as indium [14], h-BN [15], HfO_2_ [16], and In_2_O_3_ [17]. However, the hysteresis of InSe FET can hardly be eliminated, which also degrades the performance of logic devices. According to the properties of InSe materials, the decay of materials and FETs in air environment is studied, which is necessary for device process improvement and promoting performance.

In this work, we characterized InSe materials and FETs, finding that the degradation of InSe is unstoppable, but it can be mitigated through the passivation of h-BN on the top of InSe film. A high-performance InSe field effect transistor without hysteresis was fabricated using a modified method, demonstrating the great application prospect of InSe field effect transistors in integrated circuits.

## 2. Materials and Methods

In the device fabrication, a p-type Si wafer (8-inch) was cleaned with the standard clean method to remove the intrinsic silicon oxide layer on the surface and was used as the substrate, which also served as the back-gate electrode of the InSe FET. The back-gate dielectric of 20 nm HfO_2_ film was deposited by atomic layer deposition (ALD) with Hf[N(C_2_H_5_)CH_3_]_4_ (TEMAH) and H_2_O as Hf and oxygen precursors. The injection sequence of the reaction chamber is TEMAH-_2_-H_2_O-N_2_ in which N_2_ is purge gas and its purpose is to remove the excess gas and other by-products. Next, the rapid thermal annealing (RTA) process was implemented at 450 ℃ for 15 s to improve the dielectric film quality [18].

The InSe flakes were obtained by mechanical exfoliation using Scotch tape. Firstly, the bulk InSe (purchased from Six Carbon Technology) was thinned using tape. Then, the tape was attached to the target substrate and torn off to complete InSe flakes transfer. After that, we used lithography to define the source and drain contact region and Ti/Au (10 nm/40 nm) were deposited by electron beam evaporation. Finally, an atomic force microscope (AFM) was used to characterize the thickness of InSe and the electrical properties were tested by Keithley 4200 in an atmospheric environment. Raman measurements were carried out using a confocal Raman microscope with a 100× objective and a Si detector. The measurements were done using a 532 nm excitation laser source in an ambient environment at room temperature. The laser power was maintained under 1 mW to avoid any local heating.

## 3. Results and Discussion

**Figure 1:**
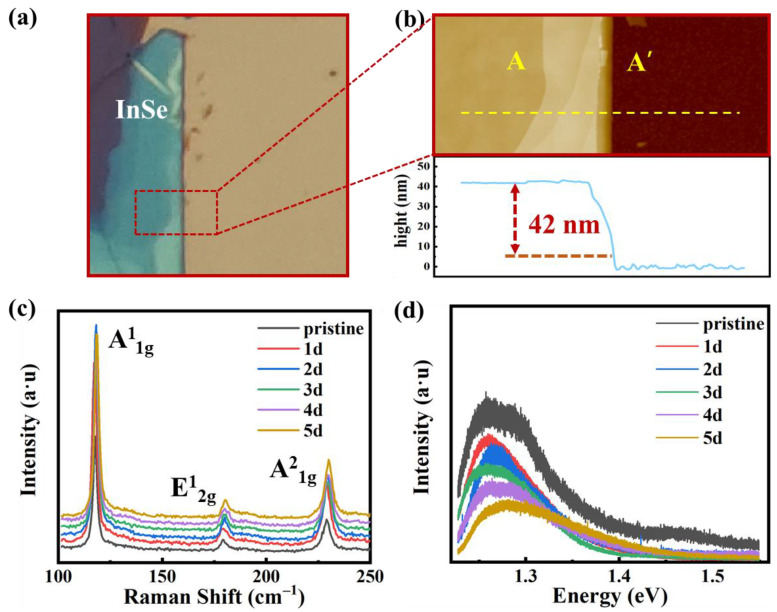
(**a**) Photo image of exfoliated InSe flake on substrate of HfO_2_/Si and the thickness is tested in the dash line area. (**b**) AFM result of thickness along AA’ and the thickness is 42 nm, corresponding to the green InSe flakes. (**c**,**d**) The Raman and PL spectra of 42 nm InSe flake preserved for 1 day, 2 days, 3 days, 4 days, and 5 days, respectively.

In this paper, we used exfoliated InSe as the channel of FETs and the variation optical contrast in microscope images of InSe flakes transferred on HfO_2_/Si substrate are shown in Figure 1a. As the MoS_2_ flakes prepared on SiO_2_/Si substrate [19], InSe films, prepared on HfO_2_/Si substrate, have different kinds of colors reflecting under the optical microscope, which can be related to nanometric changes in the thickness of films. Atomic force microscopy (AFM) results of Figure 1b show that the InSe flake is 42 nm thick with a color of light green, indicating that can be univocally identified with the color contrast areas observed in the optical image. Then, the Raman spectra and PL (Photoluminescence) spectra were both implemented with 532 nm excitation laser source at room temperature. As shown in Figure 1c, three peaks come from vibration modes of A^1^_1g_, E^1^_2g_, and A^2^_1g_ were obviously observed at 117.7 cm^−1^, 179.3 cm^−1^, and 229.1 cm^−1^, respectively. Other 21 vibrational modes are forbidden by Raman selected rules, relatively weak or degenerated [20]. With the extension of preservation time, no drift occurred in Raman peaks, indicating that the vibration mode is not affected by external factors. As shown in Figure 1d, peaks of PL spectra shift from 1.25 to 1.28 eV, demonstrating that the InSe flake is getting thinner, corresponding to the previous study showing that InSe changes from direct to indirect band gap material as the decrease of thickness [21]. The slight shift in PL peaks of 0.03 eV suggests a few layers decrease, which may be due to the highly oxidized properties of InSe film in air environment.

**Figure 2:**
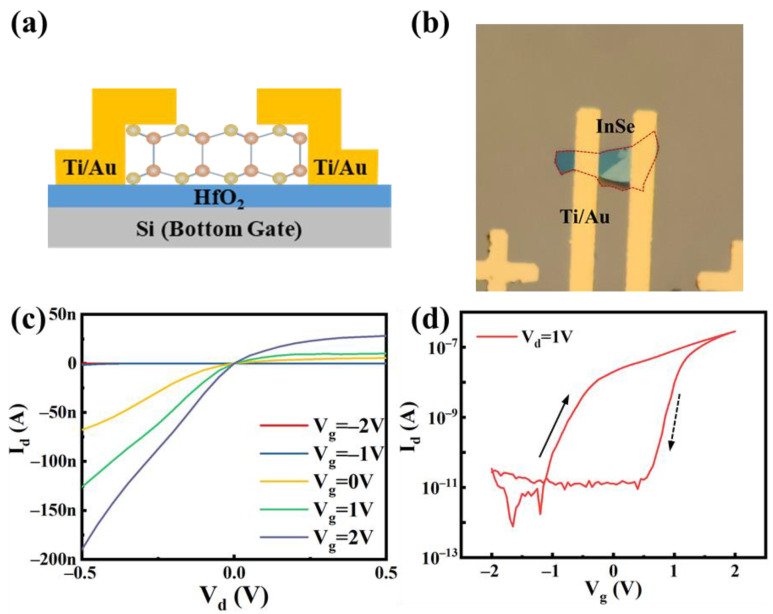
(**a**) Schematic diagram of bottom-gated InSe FET. (**b**) Optical microscope image of a typical InSe FET. (**c**,**d**) Transfer and output transport characteristic curves of InSe FET. Output curves are obtained at V_g_ = −2 V to V_g_ = 2 V, with a step of 1 V, and transfer curves are measured at V_d_ = 1 V. The solid and dash arrows represent Vg scans from −2 V to 2 V and from 2 V to −2 V.

Schematic diagram and image under an optical microscope of the fabricated device are shown in Figure 2a,b. The asymmetric output curves described in Figure 2c suggested that the contact resistance is asymmetric, which is attribute to the non-ideal interface between metal and 2D InSe. The fabrication process could have a great impact on contact quality such as photo resist organic residues [22], and material damage caused by high-energy metal deposition processes [23]. Figure 2d shows the I_d_-V_g_ transport characteristics of the device at V_d_ = 1 V. As the V_g_ changed from −2 V to 2 V with a step of 0.05 mV, I_D_ increased from 10^−12^ A to 10^−7^ A, showing the typical behavior of n-type semiconductor. With the change of gate voltage sweep direction, the current hysteresis appears, which indicates that there are many impurities and defects on the surface of 2D InSe-channel and charge traps are formed. The processes of charge and release in charge traps are responsible for the discoincidence of transfer characteristic curves when scanning direction changes. Besides, InSe is easily oxidized and sensitive to the processes of solution treatment. As a result, the maximum I_on_ is only 2.8 × 10^−7^ A, far from achieving the desired performance for InSe FET [24].

**Figure 3:**
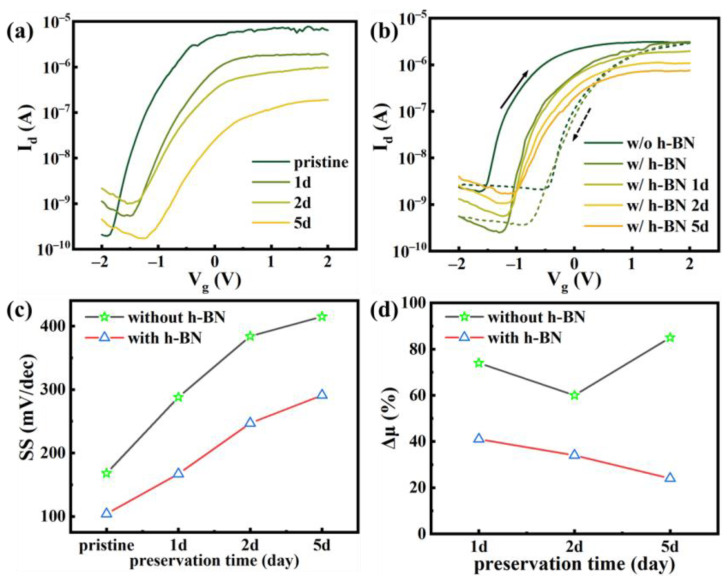
(**a**) Deterioration of electrical properties of 2D InSe FET with preservation time. (**b**) Deterioration of electrical properties after capped with h-BN. The dash lines represent the transfer curves of Vg scanning from 2 to −2 V. Different arrows indicate the direction of scanning. (**c**) Recession trend of SS for devices with and without h-BN. (**d**) Change curve of device Δμ.

To investigate the degradation of 2D InSe FET, the devices are preserved in ambient for 1 day, 2 days, and 5 days, respectively. The corresponding transfer characteristic is shown in Figure 3a. As the preservation time extended, I_on_ decreases from 7.8 × 10^−6^ A to 2 × 10^−7^ A. It is due to the increase of defects on the channel surface formed by the adsorbed impurities and oxidations, causing most of the current to scatter. V_th_ shifts from −1.2 V to −0.2 V, suggesting that traps are formed on the channel surface. These results demonstrate that the preservation in ambient would bring drastic recession to device performance. After capping with h-BN, shown in Figure 3b, I_on_ decreased from 3 × 10^−6^ A to 7.6 × 10^−7^ A and V_th_ shifted 0.1 V. The decay degree of the device is greatly reduced. Comparing the hysteresis of InSe FET with and without h-BN, it is obvious that the passivated FET has a smaller hysteresis window. After capping by h-BN, impurities on the channel surface are reduced. In Figure 3c, changing curves of subthreshold swing (SS) of InSe FET with and without h-BN are depicted. Cap-layer of h-BN can effectively decrease SS and degradation speed of SS. The rate of decline in mobility manifests the same inference, as shown in Figure 3d. These results manifest that the degradation of 2D InSe FET can be reduced by h-BN cap-layer, which is mainly due to the reduced absorption of impurities and air molecules. However, the already occurred oxidation is irreversible, and it is responsible for the degradation after passivation by h-BN. 

**Figure 4:**
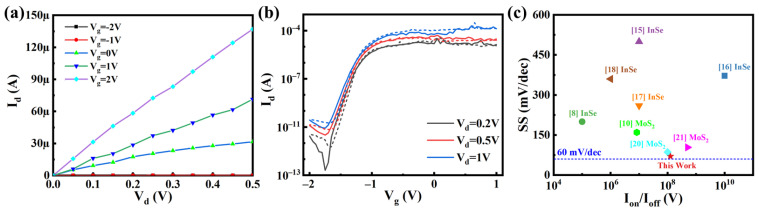
Output characteristic curves (**a**) and transfer characteristic curves (**b**) of non-lithographed device. The solid and dotted lines represent voltage scanning from −2 to 2 V and from 2 to −2 V, respectively. (**c**) Benchmark of SS and on/off ratio of transistors in different studies, including research on InSe and MoS_2_.

In order to obtain a better surface of 2D InSe FET channel, another device fabrication method is applied that we use copper hard shadow mask to define S/D contact area after InSe flakes are transferred on substrate, and then metal electrodes (Ti/Au = 10 nm/40 nm) are formed by e-beam evaporation. By using a non-photolithograph process, organic residues in contact interface are avoided and the exposure to air environment is reduced to minimize the oxidation of InSe. As a result, a high-performance InSe FET is achieved, whose output and transfer characteristic curves are described in Figure 4a,b. The nearly linear output characteristic curves demonstrate that a nearly ohmic contact is realized. According to Figure 4b, a high on/off ratio of 1.25 × 10^8^ is achieved with a maximum I_on_ of 1.98 × 10^−4^ A at Vd = 1 V. The extracted ΔVth in linear coordinates is less than 0.1 V at V_d_ = 0.2, 0.5, and 1 V, indicating that an ideal channel surface is formed for this device. In addition, this device can achieve a record low SS of 70.4 mV/dec, as we know. Finally, a benchmark of SS and I_on_/I_off_ is depicted to compare the performance of field effect transistors fabricated using InSe and MoS_2_ [10,16,17,24,25,26,27,28]. According to Figure 4c, our work demonstrates the lowest SS closing to the theoretical limits of 60 mV/dec while maintaining a relative high on/off ratio. In addition, our work not only shows a significant performance improvement in similar studies of InSe FET, but is comparable to the results of high-performance MoS_2_ FET.

## 4. Conclusions

In this work, we investigate the degradation of InSe film and InSe FET in ambient. The results of Raman, PL spectra, and electronic properties demonstrate that InSe film is easy to absorb impurities and be oxidized in ambient so that the device performance has a drastic decline. With a hard shadow mask, the device can achieve high performance with on/off ratio of 1.25 × 10^8^ and I_on_ of 1.98 × 10^−4^ A. Meanwhile, a record low SS of 70.4 mV/dec and hysteresis of 0.1 V is achieved, as we know. Our results demonstrate the huge potential of 2D InSe FET for high-performance logic devices and digital integrated circuits.

## Data Availability

The data presented in this study are available on request from the corresponding author.
